# The Significant Enhancing Effect of Vitamin B_6_-Fortified Feed on the Intestinal Digestive Efficiency, Immunity, and Antioxidant Defense Mechanisms of Juvenile Largemouth Bass (*Micropterus salmoides*)

**DOI:** 10.3390/antiox14030313

**Published:** 2025-03-05

**Authors:** Leimin Zhang, Dongyu Huang, Jiaze Gu, Hualiang Liang, Mingchun Ren

**Affiliations:** 1 Wuxi Fisheries College, Nanjing Agricultural University, Wuxi 214081, China; 2 Key Laboratory of Integrated Rice-Fish Farming Ecology, Ministry of Agriculture and Rural Affairs, Freshwater Fisheries Research Center, Chinese Academy of Fishery Sciences, Wuxi 214081, China

**Keywords:** vitamin B_6_, largemouth bass (*Micropterus salmoides*), intestine, digestion, immunity, antioxidant

## Abstract

A 12-week aquaculture trial was conducted to evaluate the effects of vitamin B_6_ on the intestinal health of largemouth bass (*Micropterus salmoides*). Six feeds with a vitamin B_6_ content of 2.03 (control group), 2.91, 3.30, 6.03, 9.53, and 21.79 mg/kg were prepared. The results were as follows. Regarding digestive efficiency, the 9.53 mg/kg group showed significantly higher activities of AMY, LPS, and TRY compared to the control group; the 6.03 mg/kg group exhibited increased AKP and Na^+^/K^+^ ATPase activities. Regarding immunity, the 6.03 mg/kg group had markedly higher relative expressions of *zo-1* and *occ* than the control group; the 9.53 mg/kg group showed significantly higher relative expressions of *il-10*, *tgf-β*, *igm*, and *cd83*, while *il-8* and *tnf-α* were notably lower, and *nf-κb* was noticeably decreased in 21.79 mg/kg group. For antioxidant capacity, the 6.03 mg/kg group had markedly higher activities of CAT, SOD, GSH-Px, and T-AOC levels, compared to the control group; the MDA level in the control group was markedly higher than in the other groups. The relative expressions of *nrf2*, *cat*, *Cu-Zn sod*, and *gpx* were highest in 9.53 mg/kg group and significantly higher than in the control group. In conclusion, an appropriate level of vitamin B_6_ in the feed is vital for protecting the intestinal health of largemouth bass.

## 1. Introduction

The intestine plays a crucial role in the physiology of fish, and it consists of the epithelial barrier, immune cells, and intestinal bacteria, which form a complex dynamic ecosystem with vital roles such as the digestion and absorption of food and the enhancement of infection resistance [1,2]. Digestion and absorption are the basic physiological functions of the intestine, efficient digestion and absorption is a direct sign of intestinal health [3]. Digestive enzyme activity acts as a key indicator for evaluating the digestive function of fish, such as amylase (AMY), lipase (LPS), and trypsin (TRY), and their functioning facilitates the efficient absorption of nutrients in the intestine [4]. The physical barrier mainly refers to the intestinal mucosal epithelium, which can effectively prevent endotoxins, antigens, and harmful microorganisms in the intestinal lumen from entering the lamina propria or intestinal mucosal capillaries during the intestinal digestive and absorption processes and then spreading to the tissues and organs outside the intestinal lumen, which is also the organism’s first line of defense against exogenous pathogenic bacteria [5,6]. In addition, as one of the largest immune organs in fish, the intestine also performs important immune and antioxidant functions, which are particularly important for improving fish immunity and disease resistance [7], and the antioxidant defense system can protect cells and tissues from oxidative damage by scavenging reactive oxygen species (ROS) and free radicals, thereby maintaining cellular function and overall health [4]. With the rapid development of aquaculture, both the environmental deterioration of the water environment caused by the intensive aquaculture model and the adjustment of feed formulations have affected the health of the fish intestine, which in turn affects the health of the fish and the outcome of the aquaculture [8,9,10]. Therefore, in view of the significant functions and vulnerability of the intestine, keeping the intestines healthy is of vital importance in upholding the health status of fish.

Nutritional regulation is the method of improving animal health through the action of nutrients in feed, which is also one of the prominent methods in protecting the intestine of aquatic animals. Studies have shown that the addition of 0.03% sodium butyrate to grass carp (*Ctenopharyngodon idella*) feeds could improve their intestinal TRY and AMY activities [11]; the appropriate amount of isoleucine in feeds improved the intestinal physical barrier function of hybrid catfish (*Pelteobagrus vachelli* × *Leiocassis longirostris*) [12] and the addition of xanthophylls in feeds could effectively alleviate the intestinal permeability, inflammation, and immune dysfunction of blunt snout bream (*Megalobrama amblycephala*) caused by diets that are high in fat and carbohydrates [13]. Vitamins, as coenzymes and active ingredients, have a crucial impact on improving the intestinal health status of aquatic animals [14,15]. For example, feed enriched with vitamin C was able to enhance the digestive and absorptive function of Chinese giant salamander (*Andrias davidianus*) [16] and Roho labeo (*Labeo rohita*) [17] through enhancing the activity of intestinal digestive enzymes, and the addition of vitamin E effectively enhanced the immunity and antioxidant ability of Roho labeo [18] and silver sillago (*Sillago sihama*) [19].

Vitamin B_6_, a water-soluble vitamin, exists primarily in three forms: pyridoxine, pyridoxal and pyridoxamine. It is closely related to the metabolism of nutrients in living organisms and is an essential nutrient for the maintenance of organismal health [20,21,22]. Studies have shown that vitamin B_6_ is equally effective in maintaining and enhancing the digestive capacity, immunity, and antioxidant function of the animal intestine. The weekly addition of 30–40 mg/L of vitamin B_6_ in water was able to increase protease activity in the intestine of Nile tilapia (*Oreochromis niloticus*) [23] and the addition of 7.47 mg/kg of vitamin B_6_ to feed was able to significantly enhance digestive enzyme and brush border enzyme activities in the intestine of golden pompano (*Trachinotus ovatus*) [24]. Lack of vitamin B_6_ in the feed reduced the content of innate immune components as well as adaptive immune components in the intestine of grass carp, which ultimately led to impairment of immune function [25]. In a study on rats, vitamin B_6_ was capable of down-regulating the mRNA expression levels of inflammatory factors and alleviating inflammatory injury [26]. In terms of antioxidant effects, the addition of vitamin B_6_ to feed at 4.96–8.58 mg/kg could considerably lessen the malondialdehyde (MDA) level and enhance the activities of antioxidant enzymes such as SOD, CAT, and GSH-Px in the intestine of Jian carp (*Cyprinus carpio* var. Jian) [27]. Furthermore, the antioxidant enzymes activities and glutathione (GSH) level were significantly reduced in rats deficient in vitamin B_6_, whereas MDA content was significantly elevated, indicating that vitamin B_6_ deficiency leads to weakened antioxidant defenses and increased oxidative stress [28]. Thus, vitamin B_6_, an essential vitamin for animals, has tremendous potential for maintaining and enhancing intestinal health in fish.

Largemouth bass, *Micropterus salmoides*, is a species of freshwater carnivorous fish, originating in inland waters in North America, that was introduced to China in the 1970s [29]. Because of its fast growth, strong adaptability, short reproduction cycle and tasty meat, it has been well appreciated by aquaculturists and consumers, its aquaculture scale has expanded rapidly, and now it has become a major freshwater aquaculture carnivorous fish species in China and globally [30,31,32]. Based on statistics, the annual production of largemouth bass in China exceeded 880,000 tons in 2023, with a year-on-year increase of more than 10%, indicating that largemouth bass occupies an important position among aquaculture species in China [33]. However, largemouth bass also face damage to their intestinal health during growth caused by environmental deterioration due to intensive aquaculture and anti-nutritional factors in feeds. For example, high levels of ammonia nitrogen in water will lead to elevated ammonia concentrations in the blood and tissues of fish, an imbalance of osmotic pressure, and ultimately damage to intestinal tissues; deteriorating water quality can lead to the proliferation of harmful microorganisms such as bacteria and viruses, which can invade the intestines of fish and cause intestinal diseases, which seriously affects their growth performance and the production efficiency of the industry, and restricts the further development. Therefore, in order to facilitate sustainable growth and progress of largemouth bass aquaculture, this research topic used a nutritional approach to investigate the role of vitamin B_6_ in maintaining the intestinal function and health of largemouth bass, with a view to providing scientific nutritional support strategies for the aquaculture industry.

## 2. Materials and Methods

### 2.1. Diet Preparation

By referring to earlier studies [34,35], six experimental feeds were designed and produced by adding six levels of pyridoxine hydrochloride to the common feed for juvenile largemouth bass. The addition of pyridoxine hydrochloride was 0, 2, 4, 8, 16, and 32 mg/kg, separately. The accurate amount of vitamin B_6_ in the feeds was measured by Standard Testing Group Co., Ltd. (Qingdao, China) and the measured values were 2.03 (control group), 2.91, 3.30, 6.03, 9.53, and 21.79 mg/kg. The method used to balance the amino acids was based on our previous study [36]. Table 1 lists the ingredient composition and the nutrient content of the common feed. The primary protein sources were fish meal, casein, and gelatin, while fish oil and soybean oil served as the major lipid sources. The process of making the feed includes crushing, sieving, weighing, mixing as well as pelleting [37]. Firstly, according to the feed formula, the feed ingredients were crushed to make sure that they could pass through a 0.18 mm sieve, then the ingredients were blended with water and oil based on the specified proportion. The mixture was processed into pellet feed with a diameter of 2 mm using a pelletizing machine model SJPS56×2 supplied by Jiangsu Muyang Group Co., Ltd. (Yangzhou, China), and then dried naturally in a cool place away from light. Finally, the dried feed was stored at about −20 °C for the proceeding aquaculture experiment.

### 2.2. Experimental Procedure

A 12-week aquacultural experiment was designed and it was conducted in an indoor recirculating aquaculture system with temperature control and purification functions from ZHONGKEHAI Recycling Water Aquaculture System Co., Ltd. (Qingdao, China) at the Feed Observatory of the Freshwater Fisheries Research Centre of the Chinese Academy of Fisheries Sciences (CAFS) (Yixing, China). Eighteen tanks (270 L per tank) were prepared, and fish for the experiment were purchased from Chia Tai Aquatic Products Co., Ltd. (Huzhou, China). All the fish were temporarily put in the tanks for adaptation before the beginning of the aquaculture. When the fish were acclimatized, they were fasted for 24 h. Then, they were weighed, and 360 fish in good health and of average weight (1.66 ± 0.01 g) were selected and grouped into 6 clusters of 3 parallels and randomly placed in the 18 tanks. In the culture process, fish were fed at 7:30, 12:30, and 17:30 based on apparent satiety every day. The fish were weighed fortnightly, and the feeding levels were adjusted accordingly, feed was administered daily at 3% of the fish’s body weight. Determination of aquaculture water quality was carried out using a kit purchased from Sampsistemi Biochemistry and Technology Co., Ltd. (Beijing, China). The water temperature was 28 ± 2 °C, the dissolved oxygen was ≥6.5 mg/L, the ammonia nitrogen was ≤0.02 mg/L, the pH value was 7.3 ± 0.3, the nitrite was 0.2 ± 0.1 mg/L, and there was a light to dark ratio of 12/12 h.

### 2.3. Sample Collection

Upon completion of the 12-week culture experiment, the fish were fasted for 24 h and then sampled. The detailed sampling process is as follows. Three fish per tank were anesthetized with MS-222 and then dissected for their mid-intestine samples. A total of 9 samples per group were used for enzyme activity and gene expression analyses related to digestive efficiency, immune response, and antioxidant capacity. These samples were stored in a liquid nitrogen tank at −196 °C immediately after collection and transferred to −80 °C after sampling for subsequent studies.

### 2.4. Chemical Analysis

Tissue homogenates were prepared using collected mid-intestine parts of juvenile largemouth bass. This operation is performed during the determination of protein content; tissue samples were ground and centrifuged according to the procedure in the corresponding instructions, and then the supernatant was aspirated as 10% homogenization supernatant. The protein content, activities of digestive enzymes such as AMY, LPS, and TRY, brush border enzymes such as alkaline phosphatase (AKP) and sodium potassium pump (Na^+^/K^+^ ATPase), enzymes reacting to antioxidant status such as catalase (CAT), superoxide dismutase (SOD), glutathione peroxidase (GSH-Px), antioxidant such as GSH, total antioxidant capacity (T-AOC), and MDA were determined by the corresponding kits purchased from Jiancheng Bioengineering Institute (Nanjing, China). The specific measurements are shown in Table 2.

### 2.5. RNA Extraction and Real-Time PCR Analysis

RNA was extracted from fish mid-intestines using the RNA extraction reagent (Vazyme, Nanjing, China). The NanoDrop 2000 spectrophotometer was used to determine the concentration and quality of RNA. The concentrations of the RNA solutions were diluted to 60 ng/μL, and the indexes of A260/A280 were between 1.8 and 2.0, indicating that the RNA purity met the requirement. Real-time PCR (RT-PCR) was performed using the One-Step qRT-PCR SYBR Green Kit (Vazyme, Nanjing, China) on a CFX96 Touch (Bio-Rad, Hercules, CA, USA). Gapdh was chosen as the control gene for its high and stable expression, which did not significantly differ among different treatments. Table 3 lists the primer sequences used in this experiment, some of which were derived from former studies; the others were designed on Primer Premier 6.1 website. The primers were synthesized by Sangon Biotech (Shanghai) Co., Ltd. (Shanghai, China). The standard curve method was used to compute the results of RT-PCR [38].

### 2.6. Statistical Analysis

Before proceeding with statistical analysis, the Shapiro–Wilk test and Levene’s test were used to assess the normality and homogeneity of variance of the data, and the results met the requirements for further analysis. The data analysis was conducted using the statistical software SPSS 25.0. One-way ANOVA (Duncan’s test) was used to compare the differences between groups. Results are expressed as means with corresponding standard deviations. The threshold for statistical significance was set at *p* < 0.05.

## 3. Results

### 3.1. Activities of Intestinal Digestive Enzymes and Brush Border Enzymes, and Relative Expression of mRNA for Related Genes

**Figure 1:**
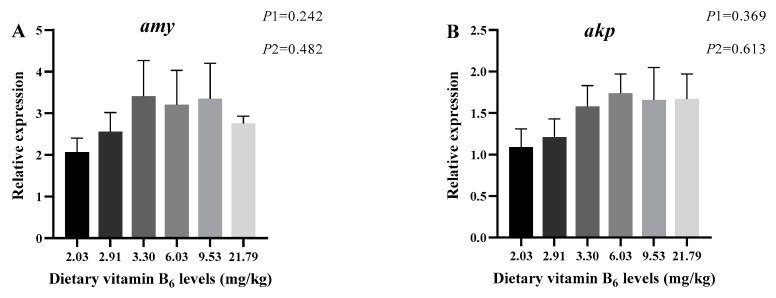
Relative expression levels of mRNA of intestinal digestion-related genes of juvenile largemouth bass fed feeds containing graded levels of vitamin B_6_ for 12 weeks. Data are mean value ± SEM (n = 3). Data with different superscript letters are significantly different (*p* < 0.05). (**A**) amy—amylase; (**B**) akp—alkaline phosphatase. *p*1: the average of the *p*-values from the Shapiro–Wilk tests for each group; *p* > 0.05 indicates that the data follow a normal distribution. *p*2: *p*-value of the Levene’s test; *p* > 0.05 indicates that the data meet the assumption of homogeneity of variances.

The results of largemouth bass intestinal digestive enzyme and brush border enzyme activities are shown in Table 4. The activities of AMS, LPS, TRY, AKP, and Na^+^/K^+^ ATPase were significantly influenced by the amount of vitamin B_6_ addition (*p* < 0.05), and all of them exhibited a trend of initially increasing and then decreasing. When compared to the control group, the activities of AMS and LPS were noticeably higher in the 9.53 mg/kg group (*p* < 0.05), TRY was remarkably higher in the 6.03 and 9.53 mg/kg groups (*p* < 0.05), AKP was remarkably higher in the 3.30 and 6.03 mg/kg groups (*p* < 0.05), and Na^+^/K^+^ ATPase was remarkably higher in the 6.03 mg/kg group (*p* < 0.05). Figure 1 illustrates the relative expression levels of *amy* and *akp*, both of which exhibited a trend of initially increasing and then decreasing; however, no significant distinctions were found among groups (*p* > 0.05).

### 3.2. Relative Expression of mRNA for Intestinal Tight Junction Proteins Genes and Immune-Related Genes

**Figure 2:**
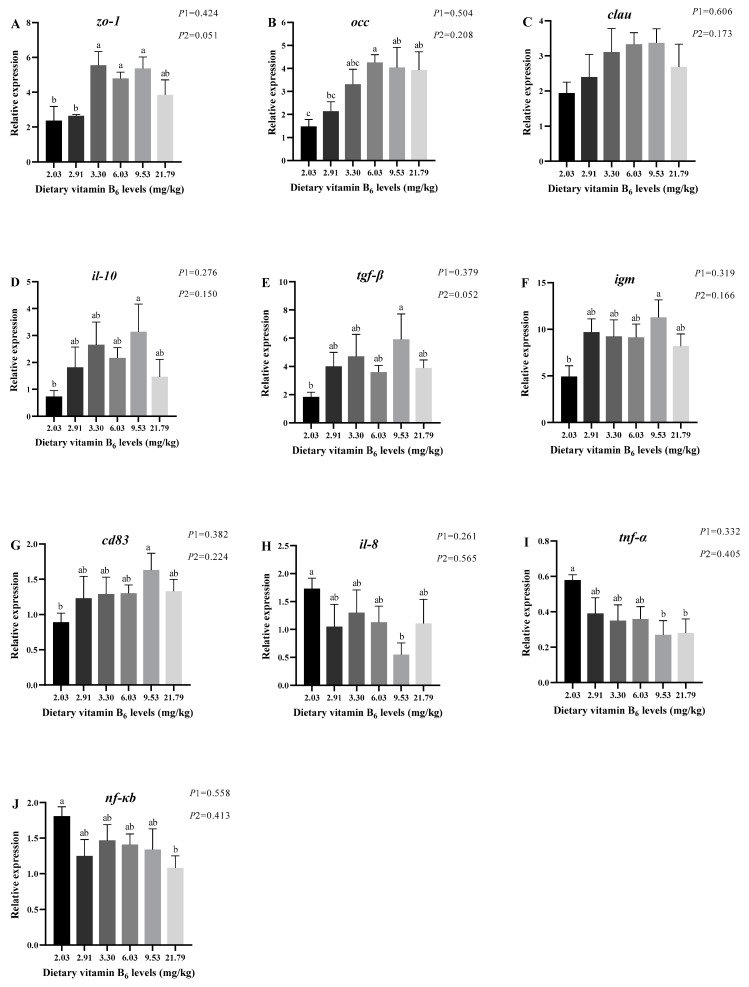
Relative expression levels of mRNA of intestinal tight junction protein genes and immune-related genes of juvenile largemouth bass fed feed with graded levels of vitamin B_6_ for 12 weeks. Data are mean value ± SEM (n = 3). Data with different superscript letters are significantly different (*p* < 0.05). (**A**) *zo-1*—zona occludens 1; (**B**) *occ*—occludin; (**C**) *clau*—claudin; (**D**) *il-10*—interleukin-10; (**E**) *tgf-β*—transforming growth factor-β; (**F**) *igm*—immunoglobulin M; (**G**) *cd83*—CD83 antigen; (**H**) *il-8*—interleukin-8; (**I**) *tnf-α*—tumor necrosis factor-α; (**J**) *nf-κb*—nuclear factor kappa-B. *p*1: the average of the *p*-values from the Shapiro–Wilk tests for each group; *p* > 0.05 indicates that the data follow a normal distribution. *p*2: *p*-value of the Levene’s test; *p* > 0.05 indicates that the data meet the assumption of homogeneity of variances.

Figure 2 shows the relative expression levels of tight junction protein genes and immunological genes of largemouth bass in different groups. As shown, the relative expression levels of intestinal tight junction protein genes, anti-inflammatory factor genes, and immunoglobulin genes exhibited a general trend of increasing and then decreasing. Compared with the 2.03 mg/kg group, the relative expression level of *zo-1* was substantially higher in the 3.30, 6.03 and 9.53 mg/kg groups (*p* < 0.05), and *occ* was considerably higher in the 6.03 mg/kg group (*p* < 0.05); *clau* did not show any significant difference among groups (*p* > 0.05). *il-10*, *tgf-β*, *igm*, and *cd83* had the highest levels in 9.53 mg/kg group and were all considerably elevated compared to the 2.03 mg/kg group (*p* < 0.05). The relative expression levels of pro-inflammatory factors exhibited a decreasing trend. In comparison with the control group, *il-8* was significantly lower in the 9.53 mg/kg group (*p* < 0.05), *tnf-α* was remarkably lower in the 9.53 and 21.79 mg/kg groups (*p* < 0.05), and *nf-κb* was significantly lower in the 21.79 mg/kg group (*p* < 0.05).

### 3.3. Activities of Antioxidant-Related Enzymes, the Levels of T-AOC, GSH, and MDA, and Relative Expression of mRNA for Antioxidant-Related Genes

**Figure 3:**
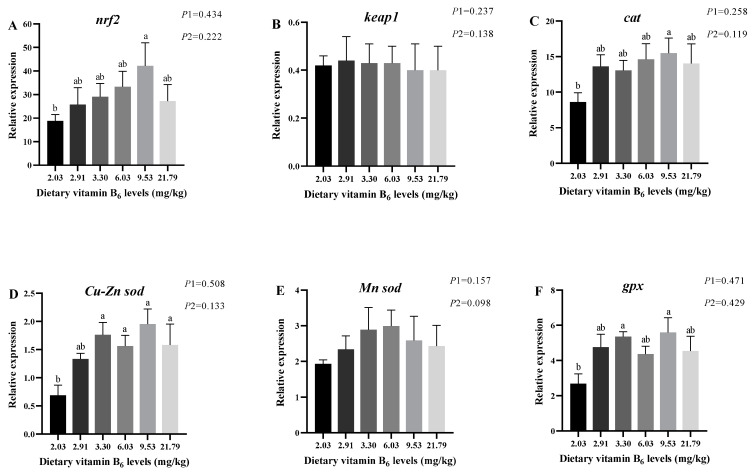
Relative expression levels of mRNA of antioxidant-related genes of juvenile largemouth bass fed graded feeds of vitamin B_6_ for 12 weeks. Data are mean value ± SEM (n = 3). Data with different superscript letters are significantly different (*p* < 0.05). (**A**) *nrf2*—nuclear factor erythroid-2-related factor2; (**B**) *keap1*—kelch-like ECH-associated protein 1; (**C**) *cat—*catalase; (**D**) *Cu-Zn sod*—Cu-Zn superoxide dismutase; (**E**) *Mn sod*—Mn superoxide dismutase; (**F**) *gpx*—glutathione peroxidase. *p*1: the average of the *p*-values from the Shapiro–Wilk tests for each group; *p* > 0.05 indicates that the data follow a normal distribution. *p*2: *p*-value of the Levene’s test; *p* > 0.05 indicates that the data meet the assumption of homogeneity of variances.

The activities of antioxidant-related enzymes, and the levels of T-AOC, GSH, and MDA in the intestine of largemouth bass are shown in Table 5. The activities of CAT, SOD, and GSH-Px, and the levels of T-AOC and GSH demonstrated an overall tendency to increase and subsequently decrease. Compared to the 2.03 mg/kg group, the activities of SOD and GSH-Px were considerably elevated in the 3.30 mg/kg group (*p* < 0.05), CAT, SOD, GSH-Px, and the levels of T-AOC were elevated in the 6.03 mg/kg group (*p* < 0.05), and GSH-Px was elevated in the 9.53 mg/kg group (*p* < 0.05). Intestinal MDA levels in largemouth bass were significantly lower in all groups supplemented with vitamin B_6_ than in the control group (*p* < 0.05). Differences in GSH levels among groups were not significant (*p* > 0.05). As for the relative expression of antioxidant-connected genes, Figure 3 shows the results. The relative expression levels of *nrf2*, *cat*, *Cu-Zn sod*, *Mn sod*, and *gpx* showed an overall increasing trend and *nrf2*, *cat*, *Cu-Zn sod*, and *gpx* reached the maximum in the 9.53 mg/kg group and were considerably higher than those in the control group (*p* < 0.05). In addition, the relative expression levels of *Cu-Zn sod* in the 3.30, 6.03, and 21.79 mg/kg groups were also noticeably higher than that in the 2.03 mg/kg group (*p* < 0.05). *Keap1* and *Mn sod* did not differ significantly among the groups (*p* > 0.05).

## 4. Discussion

### 4.1. Effect of Vitamin B6 on Intestinal Digestibility of Largemouth Bass

Digestion and absorption are the basic ways for fish to obtain nutrients and energy [40], and the intestine is the major place for digestion and absorption [41]. This process is mainly accomplished by the chemical digestion of food by digestive enzymes, so the type and activity of digestive enzymes are the physiological basis and key factors for the breakdown and absorption of feeds in fish intestine, which is an important indicator for assessing the digestive function [42,43]. There are few studies on the effect of vitamin B_6_ on intestinal digestive enzymes in fish. One study stated that the activities of chymotrypsin and AMY in the intestine of juvenile golden pompano showed a tendency to increase and then decrease with the addition of vitamin B_6_ to the feed and were significantly enhanced in the 7.47 mg/kg addition group [24]. As well as this, the weekly addition of 30–40 mg/kg of vitamin B_6_ to the culture water could significantly increase the protease activity in the intestine of Nile tilapia, thus enhancing their digestion and absorption [23]. Pyridoxal phosphate is one of the major coenzyme active forms of vitamin B_6_, as well as a coenzyme for more than 100 enzymes including transaminases, decarboxylases, and racemases [44,45]. Similarly to the above studies, in this study, the activities of AMY and LPS in the intestine of largemouth bass were significantly increased by adding 9.53 mg/kg of vitamin B_6_ to the feed, and the activities of TRY were obviously increased by both the 6.03 mg/kg and 9.53 mg/kg additional levels, which indicated that vitamin B_6_ plays a positive role, thus adding important testimony to prove that vitamin B_6_ can regulate the digestive and absorption capacity of the fish intestine. In addition, the relative expression level of *amy* showed a similar trend to that of AMY activity, but there was no significant difference between the groups.

In addition to the direct action of digestive enzymes in the process of digestion and absorption, the cells on the brush border of the intestinal villi contain brush border enzymes that are also able to promote digestion by increasing the efficiency of digestive enzymes, among which intestinal AKP can hydrolyze mono-phospholipid to maintain intestinal mucosal barrier stability as well as the normal intestinal digestive function [46], and Na^+^/K^+^ ATPase has the ability to maintain the resting potential, regulate intra- and extracellular osmotic pressure that provides potential energy for nutrient transport [47]. In our study, the trends of these two brush border enzymes activities among groups were basically consistent with those of the three digestive enzymes, and the added level of vitamin B_6_ at 6.03 mg/kg significantly increased the activities of AKP and Na^+^/K^+^ ATPase, suggesting that vitamin B_6_ could increase the activities of brush border enzymes in largemouth bass, which maintain the function of the intestinal mucosa and facilitates the transportation of nutrients. Similar conclusions have been drawn in the study of juvenile golden pompano, in which the addition of an appropriate amount of vitamin B_6_ to the feed can effectively enhance the activity of brush border enzymes including Na^+^/K^+^ ATPase [24]. The above results suggest that vitamin B_6_ can not only directly regulate the activities of digestive enzymes but also promote the digestion and absorption of nutrients by enhancing the activity of brush border enzymes.

### 4.2. Effect of Vitamin B6 on Intestinal Immunity of Largemouth Bass

The mechanical barrier is a particularly important part of the intestinal barrier, and it is an indispensable component of intestinal immunity [15]. The tight junction proteins (*zo-1*, *occ*, and *clau*) play a crucial function in this barrier; it has been found that a reduction in them increased intestinal permeability and led to decreased intestinal defenses [48,49,50]. In other research, it was found that increased expression of intestinal tight junction protein-related genes can be used as one of the signs of enhanced intestinal barrier function [51]. In our study, by adding 6.03 mg/kg of vitamin B_6_ to the feed, the relative expression of *zo-1* and *occ* was significantly elevated, indicating that vitamin B_6_ could effectively enhance the mechanical barrier of the intestine of largemouth bass, which is conducive to reducing the invasion of harmful substances and safeguarding the normal function of the intestine, but the specific mechanism of action needs to be further studied.

Vitamin B_6_ is a key compound for maintaining the function of the innate and adaptive immune system, especially for anti-inflammatory immune responses [52]. Cytokine-mediated inflammatory responses play an important regulatory role in intestinal immunity [53], and it has been shown that vitamin B_6_ could mitigate inflammatory responses in rats’ macrophages by means of activation inhibition of the nuclear factor kappa-B (*NF-κB*) signaling pathway [54]. In the present study, the addition of 9.53 mg/kg of vitamin B_6_ to the feed considerably increased the expression of anti-inflammatory factor genes, such as *il-10* and *tgf-β*, and decreased the expression of pro-inflammatory factor genes *tnf-α* and *il-8*, whereas high expression of *tnf-α* induces the overexpression of other pro-inflammatory cytokines, which is a major contributing factor in the development of inflammatory pathologies, such as intestinal malabsorption [55,56,57]. The expression of *nf-κb* was significantly reduced upon further addition. The above results indicate that vitamin B_6_ can effectively regulate the inflammatory response in the largemouth bass intestine. Other studies show similar results, where the addition of 1.7 mg/kg of vitamin B_6_ to feed was able to significantly enhance the immunity of juvenile Jian carp [58]. Conversely, when the feed was deficient in vitamin B_6_, the relative expression of anti-inflammatory factors in grass carp intestine was down-regulated, while pro-inflammatory factors were up-regulated, resulting in decreased intestinal immunity [25]. Immunoglobulin M (IgM) is the first antibody produced by fish after antigenic stimulation and has a powerful anti-infective effect, and the amount of its content is an important indicator for evaluating the immune response of fish [59]. The CD83 antigen (*cd83*) gene contributes to immune cell activation, maturation, and homeostasis, and its encoded CD83 is a member of the immunoglobulin superfamily, which was recognized as a marker of immune cell activation [60,61]. In the present study, the addition of vitamin B_6_ at 9.53 mg/kg substantially raised the relative expression of *igm* and *cd83*, indicating that largemouth bass at this time had a relatively strong immune defense. No studies have been conducted on the effect of vitamin B_6_ on fish immunoglobulins, but in a study conducted on rats, it was found that vitamin B_6_ deficiency significantly reduced IgM activity and their ability to respond to antigenic stimuli, suggesting that their immune system was impaired [62].

In summary, 6.03–9.53 mg/kg of vitamin B_6_ levels in feeds can improve the immunity of largemouth bass by enhancing the mechanical barrier of the intestine as well as upregulating the genes expression of inflammatory and immunoglobulins.

### 4.3. Effect of Vitamin B6 on Intestinal Antioxidant Capacity of Largemouth Bass

The intestine of fish is a relatively sensitive organ which is susceptible to external environmental and dietary factors due to its direct connection to the outside world, and thus is more susceptible to oxidative stress [63,64]. Vitamin B_6_, which contains hydroxyl and amine groups on its pyridine ring, is able to react directly with reactive oxygen radicals and is considered to be an effective antioxidant [65,66]. In this study, when vitamin B_6_ was added to the feed at up to 6.03 mg/kg, the activities of CAT, SOD, GSH-Px, and the levels of T-AOC, in the intestine of largemouth bass were considerably elevated compared to the unadded group. Studies have demonstrated that antioxidant enzymes in animals serve as biomarkers of response to stress and immunity, and their activity establishes the antioxidant strength of animals, which can serve as a sign for assessing the health of fish [67]; therefore, the addition of vitamin B_6_ was effective in enhancing the antioxidant capacity of the intestine of largemouth bass. This finding is similar to other studies in which the addition of vitamin B_6_ to the feed significantly increased the intestinal antioxidant enzymes activities in Jian carp [27]. Conversely, when vitamin B_6_ was deficient in the feed, a decrease in SOD activity and higher levels of reactive oxygen species (ROS) were observed in grouper (*Epinephelus coioides*), indicating increased oxidative stress [68]. MDA is a toxic substance produced by the oxidation of polyunsaturated fatty acids in cell membranes, and the level of MDA in animals can directly reflect lipid peroxidation and cellular damage; its elevated level means biological cell damage, organism aging, and the occurrence of diseases [69,70]. Previous studies have shown that vitamin B_6_ can improve lipid metabolism in grass carp, and one of the products of lipid peroxidation, MDA, affects normal lipid metabolism by influencing the activity of related enzymes and gene expression. Therefore, one of the reasons for the improvement of lipid metabolism by vitamin B_6_ may be to enhance the antioxidant capacity of the fish body, reduce the occurrence of lipid peroxidation, lower the level of MDA, and then promote the balance of lipid metabolism [71]. In addition, studies on Pacific white shrimp (*Litopenaeus vannamei*) found that supplementation of feed with vitamin B_6_ significantly increased their GSH-Px activity and reduced the concentration of MDA, thus improving their antioxidant capacity [72]. In our experiment, the MDA content of largemouth bass intestines in each vitamin B_6_-added group was significantly lower compared to the control group, further demonstrating the function of vitamin B_6_ in alleviating oxidative stress in largemouth bass intestine.

In addition to antioxidant enzymes, antioxidant-related genes are also key indicators of the organism’s antioxidant capacity [73]. When ROS levels are elevated, *keap1*, which acts as a binding agent for *nrf2*, undergoes a conformational change that results in dissociation from *nrf2*, which is released and translocated to the nucleus and regulates the expression of downstream antioxidant enzyme genes, such as *cat*, *sod*, and *gpx*, by binding to the ARE [74,75]. Vitamin B_6_ is involved in the production of a number of biologically active substances including amino acids such as taurine and tryptophan; the existence of vitamin B_6_ is essential for their synthesis and metabolism [76,77]. Relevant studies have shown that taurine could significantly up-regulate the expression of mRNA of antioxidant-related genes through regulating the Keap1/Nrf2-ARE signaling pathway, mitigating intestinal oxidative damage and improving the intestinal health of grass carp [78] and European seabass (*Dicentrarchus labrax*) [79]. Tryptophan has similar effects [80]. The results of this experiment showed that when a certain amount (9.53 mg/kg) of vitamin B_6_ was added to the feed, the Keap1/Nrf2-ARE signaling pathway in the intestine of largemouth bass was activated, and the expression of not only nrf2, but also cat, *Cu-Zn sod*, and *gpx* were significantly elevated. Correspondingly, the expression of *keap1*, although not significantly different, also had a minimum value in the 9.53 mg/kg group, which was aligned with the changing pattern of signaling pathway activation, further demonstrating the antioxidant function of vitamin B_6_. Combined with the above findings, vitamin B_6_ could enhance the antioxidant capacity of fish by regulating the Keap1/Nrf2-ARE signaling pathway, but the specific control mechanism needs to be further investigated. The above results suggest that the addition of appropriate amounts of vitamin B_6_ to the feed could enhance the antioxidant capacity of largemouth bass by strengthening the antioxidant enzyme activities in the intestine as well as activating the expression of the Keap1/Nrf2-ARE signaling pathway.

## 5. Conclusions

Our study demonstrated that vitamin B_6_ is significantly beneficial for the intestinal health of largemouth bass. Vitamin B_6_ in feed at 6.03 mg/kg effectively boosted the activities of intestinal brush border enzymes and antioxidant enzymes, as well as the relative expression levels of intestinal tight junction proteins in largemouth bass. Furthermore, 9.53 mg/kg of vitamin B_6_ in feed significantly enhanced the activities of digestive enzymes; it also upregulated the expression of immunoglobulin-related genes by inhibiting the NF-κB signaling pathway, thereby improving immunity. In addition, 9.53 mg/kg of vitamin B_6_ in feed strengthened antioxidant capacity by activating the Keap1/Nrf2-ARE signaling pathway. In summary, vitamin B_6_ was effective in protecting and enhancing the intestinal health and functions of juvenile largemouth bass when administered at levels ranging from 6.03 mg/kg to 9.53 mg/kg.

## Figures and Tables

**Table 1:** Composition and measured nutrient contents of the common diets (% dry matter).

Ingredients	Level (%)	Ingredients	Level (%)
Fish meal ^1^	20	Vitamin premix (without vitamin B_6_) ^4^	1
Casein ^2^	28		
Gelatin ^3^	7	Mineral premix ^5^	1
Wheat Flour ^1^	16	Monocalcium phosphate	4
Fish oil	4	Microcrystalline cellulose	14.45
Soybean oil	4	Vitamin C	0.05
Choline chloride	0.5		
Analyzed proximate composition			
Crude protein (%)		46.93 ± 0.07	
Crude lipid (%)		9.45 ± 0.15	
Energy (KJ/g)		17.83 ± 0.02	

Note: ^1^ Fish meal—crude protein 67.8%, crude lipid 9.3%; wheat flour—crude protein 13.1%, crude lipid 4.0%. Both were obtained from Wuxi Tongwei Feedstuffs Co., Ltd., Wuxi, China. ^2^ The casein is hydrolysed casein without vitamins (crude protein 90.0%) obtained from Merck Drugs & Biotechnology Co., Ltd., Darmstadt, Germany. ^3^ Gelatin (crude protein 90.3%) was obtained from Shanghai Zhanyun Chemical Co., Ltd., Shanghai, China. ^4^ The vitamin premix (without vitamin B_6_) was self-configured according to the vitamin premix composition of HANOVE Biotechnology Co., Ltd. ^5^ The mineral premix was obtained from HANOVE Biotechnology Co., Ltd., Wuxi, China.

**Table 2:** The chemical analysis used in the experiment.

Items	Methods	Testing Equipment/Assay Kits
AMY ^1^	Microplate method (Model C016-1-2)	Assay kits purchased from Jiancheng Bioengineering Institute (Nanjing, China); Spectrophotometer (Thermo Fisher Multiskan GO, Shanghai, China).
LPS ^2^	Microplate method (Model A054-2-1)	
TRY ^3^	Ultraviolet colorimetry (Model A080-2-2	
AKP ^4^	Microplate method (Model A059-2-2)	
Na^+^/K^+^ ATPase ^5^	Microplate method (Model A070-2-2)	
CAT ^6^	Visible light method (Model A007-1-1)	
T-AOC ^7^	ABTS method (Model A015-2-1)	
SOD ^8^	WST-1 method (Model A001-3-2)	
GSH ^9^	Microplate method (Model A006-2-1)	
GSH-Px ^10^	Colorimetric method (Model A005-1-2)	
MDA ^11^	TBA method (Model A003-1-2)	
TP ^12^	Coomassie Brilliant Blue method (Model A045-2-2)	

^1^ AMY—amylase; ^2^ LPS—lipase; ^3^ TRY—trypsin; ^4^ AKP—alkaline phosphatase; ^5^ Na^+^/K^+^ ATPase—sodium potassium pump; ^6^ CAT—catalase; ^7^ T-AOC—total antioxidant capacity; ^8^ SOD—superoxide dismutase; ^9^ GSH—glutathione; ^10^ GSH-Px—glutathione peroxidase; ^11^ MDA—malondialdehyde; ^12^ TP—total protein.

**Table 3:** Primer sequences for RT-qPCR analysis.

Genes	Forward (5′-3′)	Reverse (5′-3′)	Primer Source
*zo-1* ^1^	ATCTCAGCAGGGATTCGACG	CTTTTGCGGTGGCGTTGG	XM_038701018.1
*occ* ^2^	GATATGGTGGCAGCTACGGT	TCCTACTGCGGACAGTGTTG	XM_038715419.1
*clau* ^3^	CCAGGGAAGGGGAGCAATG	GCTCTTTGAACCAGTGCGAC	XM_038713307.1
*akp* ^4^	GGTTTTCCGGAACAGCACAC	GGCAGTTTGTGTAGGGCTCT	XM_038715644.1
*amy* ^5^	ATGGGTGTGGCTGGATTCAG	GTCTGGTCTGGGTTGATGGG	XM_038717543.1
*il-10* ^6^	CGGCACAGAAATCCCAGAGC	CAGCAGGCTCACAAAATAAACATCT	XM_038696252.1
*tgf-β* ^7^	CACCAAGGAGATGCTGATT	CGTATGTTAGAGATGCTGAAG	XM_038693206.1
*igm* ^8^	GCGTCCTTCAGTGTTCAT	TGCTTCCTCGTCATCAAC	>MN871984.1
*cd83* ^9^	CACTGTTGTGCCTTGCTG	GGAGCCTCTTTGACCTTGT	XM_038710390.1
*il-8* ^10^	GAGGGTACATGTCTGGGGGA	CCTTGAAGGTTTGTTCTTCATCGT	[39]
*nf-κb* ^11^	CCACTCAGGTGTTGGAGCTT	TCCAGAGCACGACACACTTC	XP_027136364.1
*tnf-α* ^12^	CTTCGTCTACAGCCAGGCATCG	TTTGGCACACCGACCTCACC	XM_038710731.1
*nrf2* ^13^	CACAGCAGCAGCAGGAAAAG	AAGATGCTGCCGTCTGTTGA	XM_038720536.1
*keap1* ^14^	CGTACGTCCAGGCCTTACTC	TGACGGAAATAACCCCCTGC	XP_018520553.1
*cat* ^15^	CTATGGCTCTCACACCTTC	TCCTCTACTGGCAGATTCT	MK614708.1
*Cu-Zn sod* ^16^	GGTGTTTAAAGCCGTTTGTGTT	CCTCTGATTTCTCCTGTCACCT	XM_038708943.1
*Mn sod* ^17^	ACCATGCCACTTATGTCAACAAC	AAAGTCCCGCTTAATGGCCTC	XM_038727054.1
*gpx* ^18^	ATGGCTCTCATGACTGATCCAAA	GACCAACCAGGAACTTCTCAAA	XM_038697220.1
*gapdh* ^19^	ACTGTCACTCCTCCATCTT	CACGGTTGCTGTATCCAA	AZA04761.1

Note: ^1^ *zo-1*—zona occludens 1; ^2^ *occ*—occludin; ^3^ *clau*—claudin; ^4^ *akp*—alkaline phosphatase; ^5^ *amy*—amylase; ^6^ *il-10*—interleukin-10; ^7^ *tgf-β*—transforming growth factor-β; ^8^ *igm*—immunoglobulin M; ^9^ *cd83*—CD83 antigen; ^10^ *il-8*—interleukin-8; ^11^ *nf-κb*—nuclear factor kappa-B; ^12^ *tnf-α*—tumor necrosis factor-α; ^13^ *nrf2*—nuclear factor erythroid-2-related factor2; ^14^ *keap1*—kelch-like ECH-associated protein 1; ^15^ *cat*—catalase; ^16^ *Cu-Zn sod*—Cu-Zn superoxide dismutase; ^17^ *Mn sod*—Mn superoxide dismutase; ^18^ *gpx*—glutathione peroxidase; ^19^ *gapdh*—glyceraldehyde-3-phosphate dehydrogenase.

**Table 4:** Intestinal digestive enzymes and brush edge enzymes of juvenile largemouth bass fed with feeds containing six levels of vitamin B_6_ for 12 weeks.

Parameters	Dietary Vitamin B_6_ Levels (mg/kg)						*p*1	*p*2
	2.03	2.91	3.30	6.03	9.53	21.79		
AMS ^1^ (U/mgprot)	0.70 ± 0.09 ^b^	0.75 ± 0.10 ^ab^	0.95 ± 0.09 ^ab^	0.83 ± 0.16 ^ab^	1.07 ± 0.09 ^a^	0.93 ± 0.09 ^ab^	0.516	0.053
LPS ^2^ (U/gprot)	8.83 ± 0.96 ^c^	11.85 ± 0.96 ^bc^	15.52 ± 1.13 ^ab^	15.34 ± 0.73 ^ab^	18.01 ± 1.57 ^a^	14.48 ± 1.92 ^ab^	0.198	0.151
TRY ^3^ (U/mgprot)	40.77 ± 7.26 ^b^	56.38 ± 7.68 ^ab^	46.74 ± 1.61 ^ab^	71.91 ± 10.60 ^a^	68.48 ± 9.38 ^a^	57.50 ± 9.09 ^ab^	0.497	0.254
AKP ^4^ (μmol/gprot)	0.57 ± 0.04 ^b^	0.66 ± 0.03 ^ab^	0.75 ± 0.03 ^a^	0.70 ± 0.04 ^a^	0.66 ± 0.03 ^ab^	0.67 ± 0.04 ^ab^	0.368	0.601
Na^+^/K^+^ ATPase ^5^ (U/mgprot)	0.71 ± 0.12 ^b^	0.83 ± 0.31 ^ab^	1.44 ± 0.29 ^ab^	1.65 ± 0.25 ^a^	1.03 ± 0.27 ^ab^	0.91 ± 0.25 ^ab^	0.488	0.342
TP ^6^ (g/L)	2.60 ± 0.12	2.63 ± 0.07	2.41 ± 0.13	2.49 ± 0.08	2.64 ± 0.03	2.53 ± 0.07	0.451	0.332

Note: Data are mean value ± SEM (n = 3). Means with different superscript letters in the same row are significantly different (*p* < 0.05). ^1^ AMS—amylase; ^2^ LPS—lipase.; ^3^ TRY—trypsin; ^4^ AKP—alkaline phosphatase; ^5^ Na^+^/K^+^ ATPase—sodium potassium pump; ^6^ TP—total protein. *p*1: the average of the *p*-values from the Shapiro–Wilk tests for each group; *p* > 0.05 indicates that the data follow a normal distribution. *p*2: *p*-value of the Levene’s test; *p* > 0.05 indicates that the data meet the assumption of homogeneity of variances.

**Table 5:** Antioxidant enzyme activities and the levels of T-AOC, GSH, and MDA of juvenile largemouth bass fed with feeds containing six levels of vitamin B_6_ for 12 weeks.

Parameters	Dietary Vitamin B_6_ Levels (mg/kg)						*p*1	*p*2
	2.03	2.91	3.30	6.03	9.53	21.79		
CAT ^1^ (U/mgprot)	35.56 ± 6.73 ^b^	46.66 ± 5.08 ^ab^	48.92 ± 5.31 ^ab^	56.77 ± 10.35 ^a^	48.40 ± 6.20 ^ab^	46.33 ± 3.35 ^ab^	0.397	0.425
T-AOC ^2^ (mmol/gprot)	0.17 ± 0.01 ^b^	0.26 ± 0.02 ^a^	0.24 ± 0.04 ^ab^	0.26 ± 0.02 ^a^	0.22 ± 0.03 ^ab^	0.24 ± 0.01 ^ab^	0.388	0.203
SOD ^3^ (U/mgprot)	8.21 ± 0.23 ^c^	8.74 ± 0.31 ^abc^	9.41 ± 0.34 ^ab^	9.58 ± 0.42 ^a^	8.51 ± 0.17 ^bc^	8.91 ± 0.22 ^abc^	0.512	0.205
GSH ^4^ (μmol/gprot)	163.28 ± 18.34	153.53 ± 14.66	174.92 ± 17.02	184.52 ± 10.71	177.99 ± 8.53	165.95 ± 19.78	0.286	0.239
GSH-Px ^5^ (U/mgprot)	218.44 ± 29.64 ^c^	191.82 ± 18.78 ^c^	398.74 ± 14.71 ^a^	429.35 ± 27.36 ^a^	454.01 ± 34.43 ^a^	302.96 ± 35.59 ^b^	0.598	0.221
MDA ^6^ (nmol/mgprot)	21.85 ± 2.79 ^a^	14.49 ± 1.66 ^b^	15.23 ± 1.34 ^b^	15.28 ± 1.41 ^b^	16.35 ± 0.36 ^b^	13.36 ± 1.31 ^b^	0.380	0.078
TP ^7^ (g/L)	2.60 ± 0.12	2.63 ± 0.07	2.41 ± 0.13	2.49 ± 0.08	2.64 ± 0.03	2.53 ± 0.07	0.451	0.332

Note: Data are mean value ± SEM (n = 3). Means with different superscript letters in the same row are significantly different (*p* < 0.05). ^1^ CAT—catalase; ^2^ T-AOC—total antioxidant capacity; ^3^ SOD—superoxide dismutase; ^4^ GSH—glutathione; ^5^ GSH-Px—glutathione peroxidase; ^6^ MDA—malondialdehyde; ^7^ TP—total protein. *p*1: the average of the *p*-values from the Shapiro–Wilk tests for each group; *p* > 0.05 indicates that the data follow a normal distribution. *p*2: *p*-value of the Levene’s test; *p* > 0.05 indicates that the data meet the assumption of homogeneity of variances.

## Data Availability

The data presented in this study are available on request from the corresponding author as the resultant data are contained within the article.
